# Endocrine manifestations of Luscan–Lumish syndrome associated with a novel SETD2 frameshift variant

**DOI:** 10.1210/jcemcr/luag248

**Published:** 2026-10-05

**Authors:** Julia Huang, Diana Ashouri, Alyssa Lampe Dominguez

**Affiliations:** Division of Endocrinology, Los Angeles General Medical Center and Keck Medicine of USC, Los Angeles, CA 90033, USA; Division of Endocrinology, Los Angeles General Medical Center and Keck Medicine of USC, Los Angeles, CA 90033, USA; Division of Endocrinology, Los Angeles General Medical Center and Keck Medicine of USC, Los Angeles, CA 90033, USA

**Keywords:** Luscan–Lumish syndrome, *SETD2*, genetic obesity, hypogonadotropic hypogonadism, hyperprolactinemia

## Abstract

Luscan–Lumish syndrome is a rare autosomal-dominant disorder caused by pathogenic SET domain-containing protein 2 (*SETD2*) variants, classically associated with postnatal overgrowth, obesity, developmental delay, seizures, and Chiari I malformation; endocrine manifestations remain poorly characterized. We report a 47-year-old woman with a novel *SETD2* frameshift variant (c.4451_4454del, p.Lys1486Argfs*28) presenting with severe obesity (body mass index 62.9 kg/m^2^), hypogonadotropic hypogonadism, hyperprolactinemia, and neurodevelopmental manifestations. She underwent rapid weight gain of 60 pounds during adolescence without reported dietary or activity changes, accompanied by hirsutism and secondary amenorrhea. Laboratory evaluation revealed low gonadotropins (luteinizing hormone 0.3 IU/L; reference 1.9-12.5 IU/L, follicular; follicle-stimulating hormone 0.5 IU/L; reference 2.5-10.2 IU/L, follicular), low estradiol (26.4-48 pg/mL [96.9-176.2 pmol/L]; reference 30-400 pg/mL [110.1-1468.4 pmol/L]), and hyperprolactinemia (140 ng/mL [140 µg/L]; reference 4-25 ng/mL [4-25 µg/L]). Despite decades of evaluation, a unifying diagnosis was not established until genetic testing identified the pathogenic SETD2 variant. This case expands the endocrine phenotype of Luscan–Lumish syndrome to include hypogonadotropic hypogonadism and hyperprolactinemia associated with a pituitary microadenoma, highlighting the importance of genetic evaluation in patients with rapid-onset severe obesity and multisystem disease.

## Introduction

Luscan–Lumish syndrome (LLS) is a rare neurodevelopmental disorder caused by pathogenic SET domain-containing protein 2 (*SETD2*) variants (Online Mendelian Inheritance in Man 616831) [1, 2]. It is characterized by overgrowth, obesity, developmental delay, seizures, and brain malformations. Endocrine manifestations remain incompletely described [1, 3]. We report a 47-year-old woman with a novel pathogenic *SETD2* frameshift variant whose initial presentation had been previously attributed to polycystic ovary syndrome and isolated hyperprolactinemia, thereby illustrating diagnostic pitfalls and expanding the endocrine phenotype of LLS.

## Case presentation

A 47-year-old woman was referred from neurology for evaluation of hyperprolactinemia (prolactin 140 ng/mL [140 µg/L]; reference 4-25 ng/mL [4-25 µg/L]) with a stable 3 × 2 mm nonenhancing pituitary microadenoma on magnetic resonance imaging performed at an outside institution. Original images were not available for review. Her medical history included seizures since adolescence, Chiari I malformation status postdecompression in 2012, hypertension, hypothyroidism (diagnosed at age 34 and well controlled on levothyroxine), depression, developmental delay, and longstanding secondary amenorrhea.

Menarche occurred at age 14, with early irregularity progressing to secondary amenorrhea by age 27, prompting endocrine evaluation. Hyperprolactinemia and a pituitary microadenoma were identified, and she was treated with cabergoline (0.5 mg twice weekly) for 5 years, with normalization of prolactin. She was subsequently trialed on a combined oral contraceptive pill (norgestimate/ethinyl estradiol; 0.18/0.215/0.25 mg-35 μg), with resumption of menses; however, this was discontinued shortly thereafter due to prolonged heavy menstrual bleeding lasting up to 16 days.

The patient was reportedly appropriate for gestational age in size at birth and underweight until age 14, when she underwent rapid weight gain of ∼60 pounds over 3 months, followed by progressive weight gain totaling ∼200 pounds over subsequent decades, reaching 151 kg (333 lb) at age 47. Her adult height was 154.9 cm (5 ft 1 in), below the expected midparental target height of ∼161 cm (5 ft 3.5 in), and there was no history of childhood linear growth failure. Multiple weight-loss interventions were ineffective. Metformin (500 mg twice daily) was discontinued due to perceived weight gain, and semaglutide (0.25 mg weekly) was stopped shortly following initiation due to gastrointestinal intolerance, including 1 episode of fecal incontinence.

Severe hirsutism developed in adolescence, involving the face and trunk and requiring regular mechanical hair removal. A Ferriman–Gallwey score was not documented. Spironolactone (100 mg daily for 6 months) provided minimal benefit, and total testosterone remained within reference range.

Developmentally, the patient had significant speech delay, not speaking until age 6 with longstanding learning difficulties. Formal educational evaluation in fourth grade identified multiple learning disabilities, including an auditory processing disorder, and she required special education with prolonged academic support. Autism spectrum disorder was considered but not confirmed, although the family noted behavioral features including fixation on specific activities and persistent articulation difficulties.

Seizures began at age 14, temporally overlapping the onset of rapid weight gain. Despite decades of evaluation, no unifying diagnosis had been established. The major clinical, neurologic, and endocrine events leading to diagnosis are summarized in Fig. 1. Family history was notable for obesity and polycystic ovary syndrome in a sister, estrogen receptor–positive breast cancer in her mother, and metabolic disease (diabetes in an uncle; hyperlipidemia and hypertension in both parents). Notably, neither parent had obesity or a similar phenotype.

**Figure 1:**
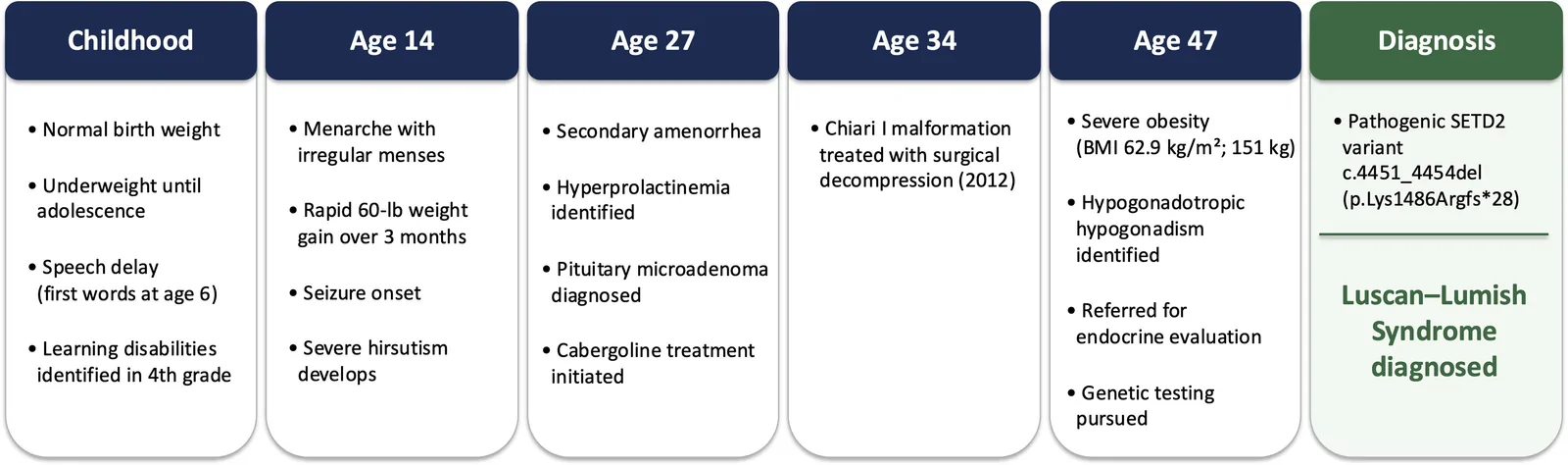
Clinical timeline of major neurologic and endocrine manifestations leading to diagnosis of LLS. Timeline showing key neurodevelopmental, neurologic, and endocrine events that emerged over more than 3 decades prior to molecular diagnosis at age 47. Abbreviation: BMI, body mass index.

## Diagnostic assessment

On examination, body mass index was 62.9 kg/m^2^ (151 kg, 154.9 cm), blood pressure 143/77 mmHg, heart rate 86 beats per min, respiratory rate 18 breaths per min. Physical examination was notable for severe obesity and marked hirsutism, with coarse terminal hair over the upper lip and chin. Her phenotype appeared distinct from that of either parent on examination (Fig. 2). There was no visible thyromegaly or proptosis; extraocular movements were intact, and cardiopulmonary examination was unremarkable. Neurologic examination revealed no gross focal deficits, and the skin was warm and well perfused.

**Figure 2:**
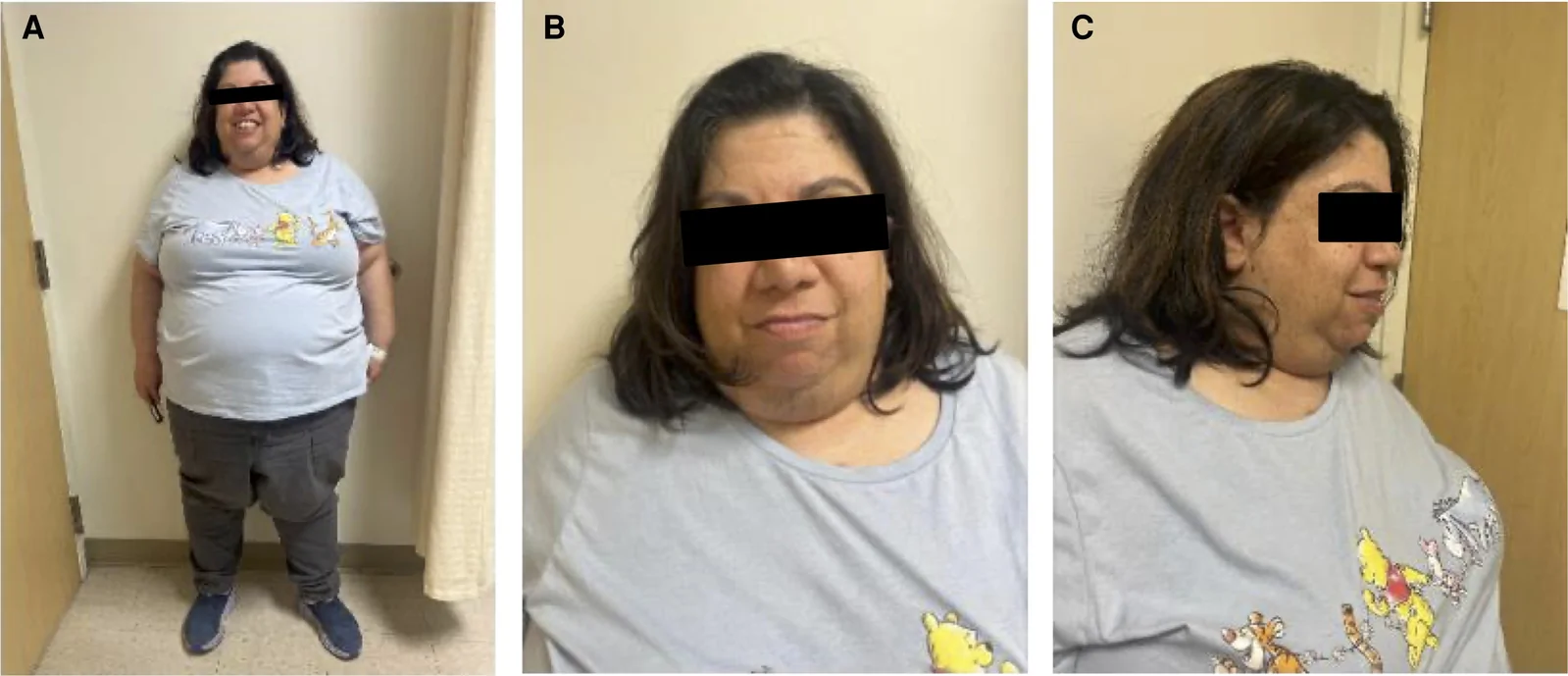
Physical examination findings in a patient with LLS. (A) Frontal view showing severe obesity with central and truncal adiposity (body mass index 62.9 kg/m^2^). (B) Frontal facial view showing facial fullness and hirsutism involving the upper lip and chin. (C) Lateral facial view showing facial profile and soft tissue fullness. Written informed consent was obtained from the patient for publication of these photographs.

Laboratory testing revealed hyperprolactinemia (140 ng/mL [140 µg/L]; reference 4-25 ng/mL [4-25 µg/L]). Gonadotropins were inappropriately low (luteinizing hormone 0.3 IU/L, follicle-stimulating hormone 0.5 IU/L) with low estradiol (26.4-48 pg/mL [96.9-176.2 pmol/L]), consistent with hypogonadotropic hypogonadism.

Total testosterone was 24 ng/dL (0.83 nmol/L; reference 8-60 ng/dL [0.28-2.08 nmol/L]), while free testosterone was mildly elevated at 5.1 pg/mL (17.7 pmol/L; reference 0.0-4.2 pg/mL [0.0-14.6 pmol/L]). Dehydroepiandrosterone sulfate was minimally elevated at 240 µg/dL (6.51 µmol/L; reference 19-231 µg/dL [0.52-6.27 µmol/L]).

Fasting insulin was elevated at 37.9 µIU/mL (227 pmol/L; reference <25 µIU/mL [<150 pmol/L]), with fasting glucose of 95 mg/dL (5.3 mmol/L; reference 70-99 mg/dL [3.9-5.5 mmol/L]). The calculated homeostatic model assessment of insulin resistance was 8.9, consistent with significant insulin resistance. Hemoglobin A1c was 5.7% (39 mmol/mol), consistent with prediabetes. Lipid studies demonstrated mixed dyslipidemia, with triglycerides 251 mg/dL (2.83 mmol/L), low-density lipoprotein cholesterol 102 mg/dL (2.64 mmol/L), and high-density lipoprotein cholesterol 38 mg/dL (0.98 mmol/L; Table 1).

**Table 1:** Endocrine and metabolic laboratory results

Parameter	Value (conventional and SI units)	Normal range (conventional and SI units)
Prolactin	140 ng/mL (140 µg/L)	4-25 ng/mL (4-25 µg/L)
LH	0.3 IU/L	1.9-12.5 IU/L (follicular)
FSH	0.5 IU/L	2.5-10.2 IU/L (follicular)
Estradiol	26.4-48 pg/mL (96.9-176.2 pmol/L)	30-400 pg/mL (110.1-1468.4 pmol/L)
Total testosterone	24 ng/dL (0.83 nmol/L)	8-60 ng/dL (0.28-2.08 nmol/L)
Free testosterone	5.1 pg/mL (17.7 pmol/L)	0.0-4.2 pg/mL (0.0-14.6 pmol/L)
DHEA-S	240 µg/dL (6.51 µmol/L)	19-231 µg/dL (0.52-6.27 µmol/L)
Androstenedione	109 ng/dL (3.80 nmol/L)	20-75 ng/dL (0.70-2.62 nmol/L) (postmenopausal)
17-Hydroxyprogesterone	11 ng/dL (0.33 nmol/L)	≤45 ng/dL (≤1.36 nmol/L) (postmenopausal)
C-peptide	3.79 ng/mL (1.25 nmol/L)	0.80-3.85 ng/mL (0.26-1.27 nmol/L)
Fasting glucose	95 mg/dL (5.3 mmol/L)	70-99 mg/dL (3.9-5.5 mmol/L)
Insulin	37.9 µIU/mL (227 pmol/L)	<25 µIU/mL (<150 pmol/L)
HOMA-IR	8.9	—
HbA1c	5.7% (39 mmol/mol)	<5.7% (<39 mmol/mol)
Triglycerides	251 mg/dL (2.83 mmol/L)	<150 mg/dL (<1.69 mmol/L)
LDL cholesterol	102 mg/dL (2.64 mmol/L)	<100 mg/dL (<2.59 mmol/L)
HDL cholesterol	38 mg/dL (0.98 mmol/L)	>50 mg/dL (>1.29 mmol/L)
IGF-1	133 ng/mL (133 µg/L)	52-328 ng/mL (52-328 µg/L)
TSH	2.1 µIU/mL (2.1 mIU/L)	0.35-4.94 µIU/mL (0.35-4.94 mIU/L)
Free T4	1.1 ng/dL (14.2 pmol/L)	0.70-1.48 ng/dL (9.0-19.1 pmol/L)

Abbreviations: DHEA-S, dehydroepiandrosterone sulfate; FSH, follicle-stimulating hormone; HbA1c, hemoglobin A1c; HDL, high-density lipoprotein; HOMA-IR, homeostatic model assessment of insulin resistance; IGF-1, insulin-like growth factor 1; LDL, low-density lipoprotein; LH, luteinizing hormone; T4, thyroxine; TSH, thyroid-stimulating hormone.

Insulin-like growth factor 1 (IGF-1) was 133 ng/mL (133 µg/L; reference 52-328 ng/mL [52-328 µg/L]), suggesting no evidence of growth hormone (GH) excess or deficiency.

Evaluation for alternative causes of hyperandrogenism included androstenedione (109 ng/dL [3.80 nmol/L]; reference 20-75 ng/dL [0.70-2.62 nmol/L], postmenopausal) and 17-hydroxyprogesterone (11 ng/dL [0.33 nmol/L]; reference ≤45 ng/dL [≤1.36 nmol/L], postmenopausal), with normal 17-hydroxyprogesterone arguing against nonclassic congenital adrenal hyperplasia. Hypercortisolism was not formally evaluated, although the clinical profile was not suggestive of Cushing syndrome. Polycystic ovary syndrome was considered but not diagnosed, as suppressed gonadotropins were not consistent with typical physiology.

Given the combination of abrupt adolescent-onset severe obesity, neurodevelopmental features (including seizures and Chiari malformation), atypical reproductive hormone profile, and phenotypic differences from both parents, a syndromic etiology was suspected. Genetic testing was pursued and revealed a novel heterozygous pathogenic frameshift variant in *SETD2*, c.4451_4454del (p.Lys1486Argfs*28; Fig. 3). The variant introduced a premature stop codon, resulting in a truncated, nonfunctional SETD2 protein; it met American College of Medical Genetics and Genomics criteria for pathogenicity and was absent from the Genome Aggregation Database (gnomAD) [4]. The loss-of-function variant and clinical phenotype supported a diagnosis of LLS [1, 2].

**Figure 3:**
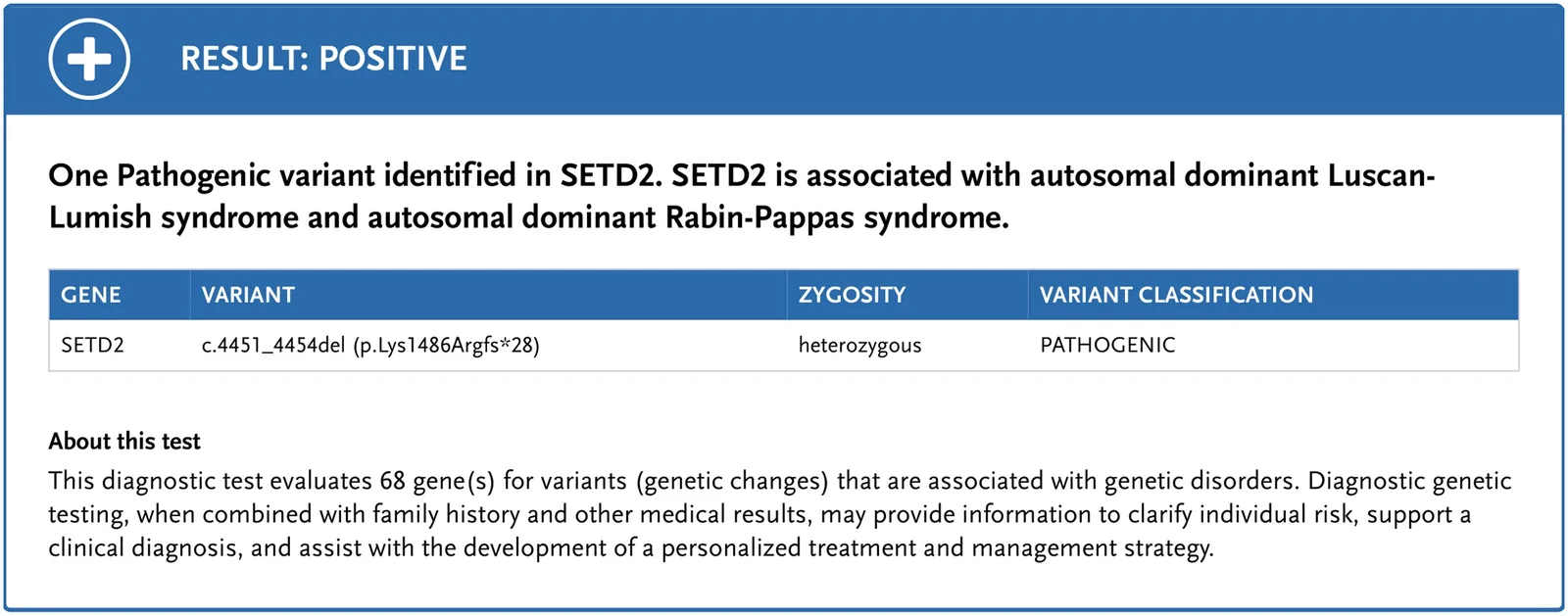
Pathogenic SET domain-containing protein 2 (*SETD2*) frameshift variant identified on genetic testing. Diagnostic genetic testing identified a heterozygous pathogenic frameshift variant in *SETD2* (c.4451_4454del; p.Lys1486Argfs*28), resulting in premature truncation of the SETD2 protein and confirming the diagnosis of LLS.

## Treatment

Following diagnosis, the patient was referred to medical genetics for counseling regarding autosomal-dominant inheritance and reproductive implications, including potential de novo origin and a 50% transmission risk to biological offspring [2]. Potential neoplasm risk was discussed [5].

For obesity and metabolic disease, tirzepatide was initiated at 2.5 mg subcutaneously once weekly with planned dose escalation every 4 weeks as tolerated. Bariatric surgery was discussed as a potential option if medical therapy proved insufficient, with counseling that outcomes in syndromic obesity may be less predictable and associated with higher rates of weight regain [6].

Given hypogonadotropic hypogonadism, hormone replacement therapy was discussed to mitigate complications of hypoestrogenism, including bone loss and cardiometabolic risk [7]. However, initiation was deferred given a maternal history of estrogen receptor–positive breast cancer, with plans for reassessment following screening mammography.

For hirsutism refractory to spironolactone, finasteride 5 mg daily was initiated with counseling that clinical improvement may require several months [8].

Hyperprolactinemia was managed conservatively given the longstanding stability of the microadenoma, prior intolerance of dopamine agonists, and lack of fertility goals, with planned surveillance [9].

## Outcome and follow-up

At last follow-up (4 months after genetic diagnosis), the patient remained under longitudinal endocrinology care, with referral to medical genetics pending. Tirzepatide was well tolerated and associated with modest weight reduction from 151 to 149 kg over 3 weeks, prompting uptitration to 5 mg weekly.

Hirsutism improved significantly after initiation of finasteride (5 mg daily), with the patient reporting a markedly greater reduction in facial hair growth compared with prior spironolactone therapy. Hyperprolactinemia remained stable without interval growth of the pituitary microadenoma, and no new seizures or neurologic complications were reported.

Following normal screening mammography, hormone replacement therapy was initiated with transdermal estradiol (0.025 mg/day) and oral progesterone (100 mg nightly). The patient remained clinically stable.

## Discussion

This case describes LLS with endocrine manifestations, including hypogonadotropic hypogonadism, hyperprolactinemia associated with a pituitary microadenoma, hirsutism with largely normal testosterone levels, and metabolic dysfunction.

*SETD2* encodes a histone methyltransferase responsible for histone H3 lysine 36 (H3K36) trimethylation, a key regulator of transcription [5, 10]. Loss-of-function variants are associated with LLS, characterized by overgrowth, obesity, developmental delay, and seizures.

Although obesity is frequently reported in LLS, endocrine characterization remains limited. In this patient, the rapid adolescent-onset weight gain—∼60 pounds over 3 months despite reported normal intake—was striking and consistent with severe, biologically driven obesity. Proposed mechanisms include hypothalamic energy dysregulation related to epigenetic disruption [10]. In 1 report, increased IGF-1 expression and signal transducer and activator of transcription 5B phosphorylation in patient-derived fibroblasts suggested enhanced GH pathway signaling [11]. As *SETD2* mediates H3K36 trimethylation, loss-of-function variants may alter expression of genes involved in GH signaling and downstream metabolic pathways. Dysregulated GH–IGF-1 signaling could, in turn, influence hypothalamic energy balance and adiposity, providing a potential mechanistic link between *SETD2* dysfunction and the rapid-onset obesity observed in this patient. Notably, serum IGF-1 was normal in our patient, suggesting that any GH–IGF-1 pathway dysregulation in LLS may occur at the tissue-specific or downstream signaling level rather than through systemic hormone excess.

Her reproductive profile was notable for low estradiol with markedly low luteinizing hormone and follicle-stimulating hormone, supporting hypogonadotropic hypogonadism. Potential contributors to hypogonadotropic hypogonadism include hypothalamic dysfunction related to neurodevelopmental abnormalities, obesity-associated functional suppression, and prolactin-mediated inhibition of gonadotropin-releasing hormone secretion [7, 9, 12]. The combination of Chiari malformation and pituitary microadenoma raises the possibility of altered hypothalamic–pituitary axis physiology, although causality cannot be established from this single case.

Hirsutism despite normal total testosterone, only mildly elevated free testosterone, and minimal elevations in adrenal androgens suggests that the severity of clinical hyperandrogenism was disproportionate to the biochemical profile. The marked improvement with finasteride, compared with prior spironolactone therapy, may support a role for increased peripheral androgen activation via 5α-reductase activity rather than overt circulating hyperandrogenemia. Hirsutism has been reported in a subset of LLS cases [3, 8].

Obesity, including severe obesity, has been previously described in LLS. To our knowledge, hypogonadotropic hypogonadism and hyperprolactinemia associated with a pituitary microadenoma have not been previously described. This case expands the recognized endocrine phenotype of LLS to include these features, as well as metabolic dysfunction and hirsutism with largely normal testosterone levels. While several features, including neurodevelopmental abnormalities, obesity, and hypogonadotropic hypogonadism, are consistent with a potential neuroendocrine manifestation of LLS, other findings such as hypothyroidism and depression may be coincidental or multifactorial, and whether the pituitary microadenoma is related to the underlying genetic disorder remains uncertain.

This case highlights the importance of genetic evaluation in patients with rapid-onset obesity and multisystem features. Further studies are needed to define the endocrine natural history and treatment response in *SETD2-*related disease.

## Learning Points

- Genetic testing is essential in patients with unexplained multisystem disease to establish a unifying diagnosis and guide management.
- Consider LLS in patients with rapid-onset severe obesity and neurodevelopmental manifestations, particularly when the clinical presentation is atypical or unexplained.
- Luscan–Lumish syndrome may present with endocrine manifestations, including hypogonadotropic hypogonadism, hyperprolactinemia associated with a pituitary microadenoma, metabolic dysfunction, and hirsutism despite largely normal androgen levels.

## Data Availability

Original data generated and analyzed during this study are included in this published article.
